# Characterization of primary human hepatocyte spheroids as a model system for drug-induced liver injury, liver function and disease

**DOI:** 10.1038/srep25187

**Published:** 2016-05-04

**Authors:** Catherine C. Bell, Delilah F. G. Hendriks, Sabrina M. L. Moro, Ewa Ellis, Joanne Walsh, Anna Renblom, Lisa Fredriksson Puigvert, Anita C. A. Dankers, Frank Jacobs, Jan Snoeys, Rowena L. Sison-Young, Rosalind E. Jenkins, Åsa Nordling, Souren Mkrtchian, B. Kevin Park, Neil R. Kitteringham, Christopher E. P. Goldring, Volker M. Lauschke, Magnus Ingelman-Sundberg

**Affiliations:** 1 Department of Physiology and Pharmacology, Section of Pharmacogenetics, Karolinska Institutet, Stockholm, Sweden; 2 Department of Clinical Science, Intervention and Technology, Karolinska University Hospital Huddinge, Karolinska Institutet, Stockholm, Sweden; 3 MRC Centre for Drug Safety Science, Department of Molecular and Clinical Pharmacology, Sherrington Buildings, Ashton Street, University of Liverpool, UK; 4 Janssen Pharmaceutical Companies of Johnson & Johnson, Department of Pharmacokinetics, Dynamics and Metabolism, Beerse, Belgium

## Abstract

Liver biology and function, drug-induced liver injury (DILI) and liver diseases are difficult to study using current *in vitro* models such as primary human hepatocyte (PHH) monolayer cultures, as their rapid de-differentiation restricts their usefulness substantially. Thus, we have developed and extensively characterized an easily scalable 3D PHH spheroid system in chemically-defined, serum-free conditions. Using whole proteome analyses, we found that PHH spheroids cultured this way were similar to the liver *in vivo* and even retained their inter-individual variability. Furthermore, PHH spheroids remained phenotypically stable and retained morphology, viability, and hepatocyte-specific functions for culture periods of at least 5 weeks. We show that under chronic exposure, the sensitivity of the hepatocytes drastically increased and toxicity of a set of hepatotoxins was detected at clinically relevant concentrations. An interesting example was the chronic toxicity of fialuridine for which hepatotoxicity was mimicked after repeated-dosing in the PHH spheroid model, not possible to detect using previous *in vitro* systems. Additionally, we provide proof-of-principle that PHH spheroids can reflect liver pathologies such as cholestasis, steatosis and viral hepatitis. Combined, our results demonstrate that the PHH spheroid system presented here constitutes a versatile and promising *in vitro* system to study liver function, liver diseases, drug targets and long-term DILI.

The liver is a vital organ for synthesis, metabolism and detoxification, but liver diseases and drug-induced liver injury (DILI) can severely impair liver functionality. To study liver biology and function, drug-induced hepatotoxicity and liver diseases, primary human hepatocytes (PHH) are currently considered as the gold standard *in vitro* model system^1^. However, when maintained in conventional 2D monolayer cultures, PHH de-differentiate and rapidly lose hepatocyte-specific functions^2^,^3^,^4^. Thus, the utility of conventional 2D PHH cultures for the long-term study of liver biology and assays that require liver-specific functionalities is largely impaired. There is therefore a need for more faithful *in vitro* models which more accurately reflect *in vivo* liver biology. To this end, new systems are needed in which stable liver functionality can be maintained for several weeks to enable long-term studies of liver function under normal and diseased conditions.

Normal cell physiology and function strongly depend on cell-cell and cell-extracellular matrix (ECM) interactions in the 3D tissue environment^5^. In an attempt to mimic the hepatic microenvironment, various more complex culture systems have been developed including sandwich cultures, and 3D models such as scaffold-based systems and bioreactors^6^,^7^,^8^,^9^,^10^,^11^,^12^. However, major drawbacks of these culture systems include lack of scalability, binding of drugs to scaffold, difficulties in handling and batch-to-batch differences of ECM substrates, which affect reproducibility^11^.

To circumvent these problems, hepatocytes can be cultured as 3D microtissues termed spheroids^13^,^14^,^15^,^16^. In spheroid culture, it has previously been shown that PHH can be maintained for longer periods of time with stable viability and production of essential molecules such as albumin and urea^14^,^15^,^16^. Furthermore, cellular polarity and formation of functional bile ducts has been described^14^. However, a full phenotypic characterization as well as a comprehensive assessment of the suitability of the PHH spheroid model in the context of studying e.g. liver diseases and chronic DILI is lacking.

Here, we have developed and extensively characterized an easily scalable 3D PHH spheroid system in serum-free, chemically-defined conditions, suitable for long-term functional and toxicological studies. Importantly, PHH spheroids closely resembled the *in vivo* liver tissue from where they originated more than spheroid cultures isolated from other donors, as determined by whole proteome analyses. Thus, inter-individual variability is maintained on a global scale. The PHH spheroids presented here remained phenotypically stable and retained morphology, viability, and hepatocyte-specific functions for culture periods of at least 5 weeks. The culture conditions allowed co-culture of PHH spheroids with non-parenchymal cells (NPCs) such as biliary cells, stellate cells and Kupffer cells and supported their long-term viability. Furthermore, liver diseases such as steatosis, cholestasis and viral hepatitis could be induced and the spheroids could predict chronic drug toxicity in particular of fialuridine, a drug which previously caused several deaths in a clinical trial while having previously passed all pre-clinical safety assessments^17^.

Combined, these results indicate that the PHH spheroid system developed here constitutes a promising and versatile *in vitro* model to study various aspects of liver function, liver disease and DILI.

## Results

### Characterization of PHH spheroid morphology and function

**Figure 1:**
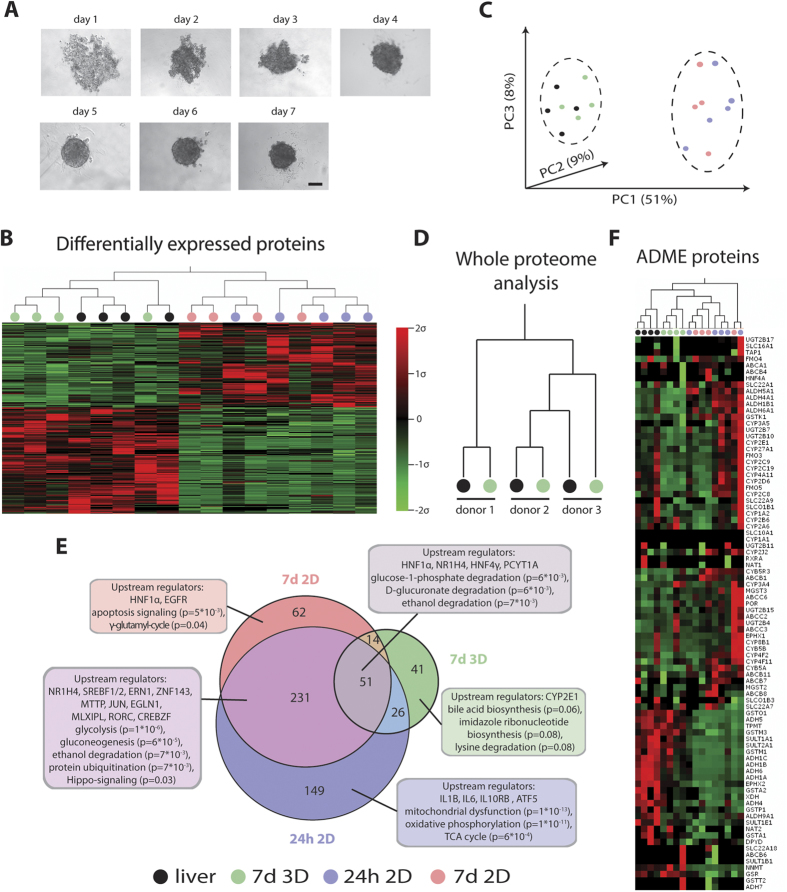
3D spheroids from PHH closely resemble the *in vivo* liver at the proteome level. (**A**) Time series showing progressing spheroid aggregation over time. Spheroid formation was judged complete after 7 d when a well-defined perimeter could be observed. Scale bar = 100 μm. (**B**) Heatmap visualizing whole proteome analysis of primary human liver samples (n = 5) after 24 h and 7 d in 2D monolayer culture and spheroids after aggregation (7 d 3D). Only differentially expressed proteins (n = 574 proteins, p < 0.05, F-test) are shown. Note that *in vivo* liver samples (black) and spheroids (green) cluster closely together while the proteomes of samples cultured in 2D (24 h = blue; 7 d = red) are distinctly different. (**C**) Principle component analysis separates proteomes from liver and PHH spheroids from 2D monolayer-cultured samples. (**D**) *In vivo* phenotypes are preserved in 3D culture, with each of the 3D samples clustering with the respective liver piece from the same donor. (**E**) Venn diagram showing differentially regulated pathways after 24 h 2D, 7 d 2D and 7 d 3D as suggested by GSEA. Numbers in circles indicate numbers of differentially expressed genes compared to liver with p < 0.05. Extensive misregulation of a variety of important metabolic and signaling pathways is observed in 2D such as glycolysis, gluconeogenesis, Hippo-signaling and apoptosis. In contrast, the proteomes of 3D PHH spheroid cultures closely resemble *in vivo* livers. Indicated p-values are after Benjamini-Hochberg multiple testing correction. (**F**) Heatmap showing all proteins involved in absorption, distribution, metabolism and excretion (ADME) of compounds that we detected in our dataset. Note that livers and PHH spheroids cultures cluster together, similar to when whole proteomes are considered.

In order to constitute a relevant hepatic *in vitro* system, cultured hepatocytes need to accurately reflect phenotypes and functionality seen *in vivo*. Therefore, PHH spheroid phenotypes and their molecular signatures were assessed and compared to freshly isolated cells from the same donor. Once spheroids had formed and showed well-defined perimeters (Fig. 1A), proteomic analyses were performed using an unbiased global proteomic approach. PHH spheroids after aggregation (7 d 3D) and cells from the same donors that were cultured in 2D as conventional monolayers (24 h 2D and 7 d 2D) were compared with the corresponding livers from which they originated (n = 5). Strikingly, it was found that proteomic signatures underwent wide-scale and rapid changes in 2D monolayer culture (Fig. 1B). Already after 24 h, expression of 457 proteins (13.9% of the entire detected proteome, p < 0.05, F-test) was significantly affected. After 7 d in 2D monolayer culture, 358 proteins were differentially expressed, of which 282 (78.8%) were already differentially expressed after 24 h.

Importantly, when the proteomes of the PHH spheroid cultures were analyzed, it was found that considerably fewer proteins were differentially expressed in spheroids (n = 132) as compared to 2D monolayer culture (n = 358). As a consequence, hierarchical clustering (Fig. 1B) as well as principal component analysis (PCA, Fig. 1C) resulted in the strict separation of liver and spheroid samples from all 2D monolayer cultures. Strikingly, 3D samples clustered together with the corresponding *in vivo* liver pieces they were derived from highlighting the preservation of inter-individual differences in PHH spheroid culture and the ability to study inter-individual variability in hepatic function in an *in vitro* system (Fig. 1D).

Next, the functional implications of the proteome alterations using gene set enrichment analyses were analyzed (GSEA, Fig. 1E). Interestingly, after multiple testing correction, we found that mitochondrial function (p = 1*10^−13^), oxidative phosphorylation (p = 1*10^−11^) and the TCA cycle (p = 6*10^−4^) were significantly affected only in the early stages of 2D culture (24 h), while the effect on a multitude of other important pathways such as glycolysis (p = 1*10^−6^), gluconeogenesis (p = 6*10^−5^), ethanol degradation (p = 7*10^−3^) and protein ubiquitination (p = 7*10^−3^) persisted after 7 d in 2D monolayer culture. Proteins involved in apoptosis signaling (p = 5*10^−3^) and the γ-glutamyl cycle (p = 0.04) were only found to be enriched after prolonged 2D culture (7 d). When analyzing the proteins that were exclusively misregulated in PHH spheroid culture, bile acid biosynthesis was found as the most enriched pathway (p = 0.06). Interestingly though, studies in rats have shown that perturbations of bile acid biosynthesis are part of the liver regeneration program in response to liver damage *in vivo*, thus suggesting that pathway perturbations seen in the PHH spheroid system closely recapitulate *in vivo* processes^18^.

Improvements to *in vitro* systems for prediction of drug metabolism and drug toxicity are needed and therefore the proteins involved in drug absorption, distribution, metabolism and excretion (ADME) were analyzed in detail. In total, 86 ADME gene products were detected in the samples. Similarly to when whole proteomes were considered, hierarchical clustering of ADME proteins revealed close similarities between PHH spheroid cultures and livers (Fig. 1F).

Combined, these results indicate that hepatic metabolism as well as intracellular signaling is highly remodeled in 2D monolayer cultures whereas in 3D spheroids, significantly fewer pathways were affected. Thus, as changes in proteomes occur very quickly even within the first 24 h, simple 2D monolayer cultures are phenotypically inferior to PHH spheroids even for short-term acute toxicity tests.

### PHH spheroids can be stably maintained in serum-free conditions for at least 5 weeks

**Figure 2:**
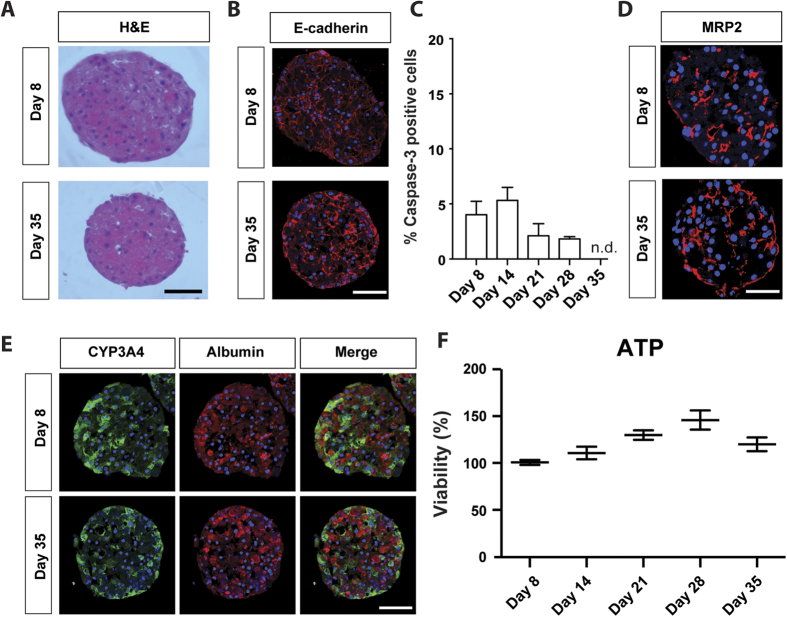
PHH spheroids can be maintained for at least 5 weeks in serum-free conditions. (**A**) H&E staining of PHH spheroids. Note that spheroid sizes decreased coinciding with increased expression of E-cadherin (**B**), which has been previously shown to promote spheroid compaction^5^. (**C**) Levels of cleaved caspase-3, a marker for apoptosis, remained at low levels throughout the culture period as determined by immunohistochemistry. (**D**) MRP2 immunostaining revealed bile canaliculi at early as well as late stages of spheroid culture. (**E**) Staining for the perivenous marker CYP3A4 and the periportal marker albumin reveals that the zonation-identity of liver cells is maintained. (**F**) Cellular ATP levels remained constant throughout 5 weeks of culture (n = 20 spheroids from 3 donors per time point). Absolute ATP values were normalized to spheroid volume to compensate for compaction. All scale bars = 100 μm.

In order to assess PHH spheroid performance in long-term experiments, spheroids were characterized both morphologically and functionally for 35 days in culture. Certain batches of PHH aggregated at lower efficiency and so a threshold for use was the requirement of good spheroid formation within 7 days. Seeding of 1,500 cells/well resulted in spheroids of a consistent size (~200 μm diameter), ensuring sufficient diffusion of nutrients such as oxygen to the core^19^. These spheroids showed homogenous morphology, clearly defined cell boundaries and intact nuclei without evident necrosis after 5 weeks in culture as revealed by H&E staining (Fig. 2A). During culture, spheroid sizes decreased coinciding with an increase in expression of the transmembrane protein E-cadherin (Fig. 2B), which has been shown to play an important role in cell-cell adhesion and spheroid compaction^5^, suggesting that spheroids become increasingly compact during the culture time. Shrinkage due to loss of hepatocytes during cell cultivation is also likely since staining for the apoptosis marker cleaved caspase-3 remained detectable, but at low levels throughout the culture period (Fig. 2C). Furthermore, MRP2 staining indicated that functional bile canaliculi were evident even after prolonged culture times (Fig. 2D).

Interestingly, immunohistochemical staining showed only marginal overlap of CYP3A4 (perivenous marker) and albumin (periportal marker) (Fig. 2E), indicating that the phenotype of cells originating from either the periportal or the perivenous region of the liver acinus is retained during long-term cultivation^20^. Cell viability was assessed by quantification of ATP levels which remained rather constant in relation to spheroid size (Fig. 2F) suggesting no decrease in viability in long-term culture.

**Figure 3:**
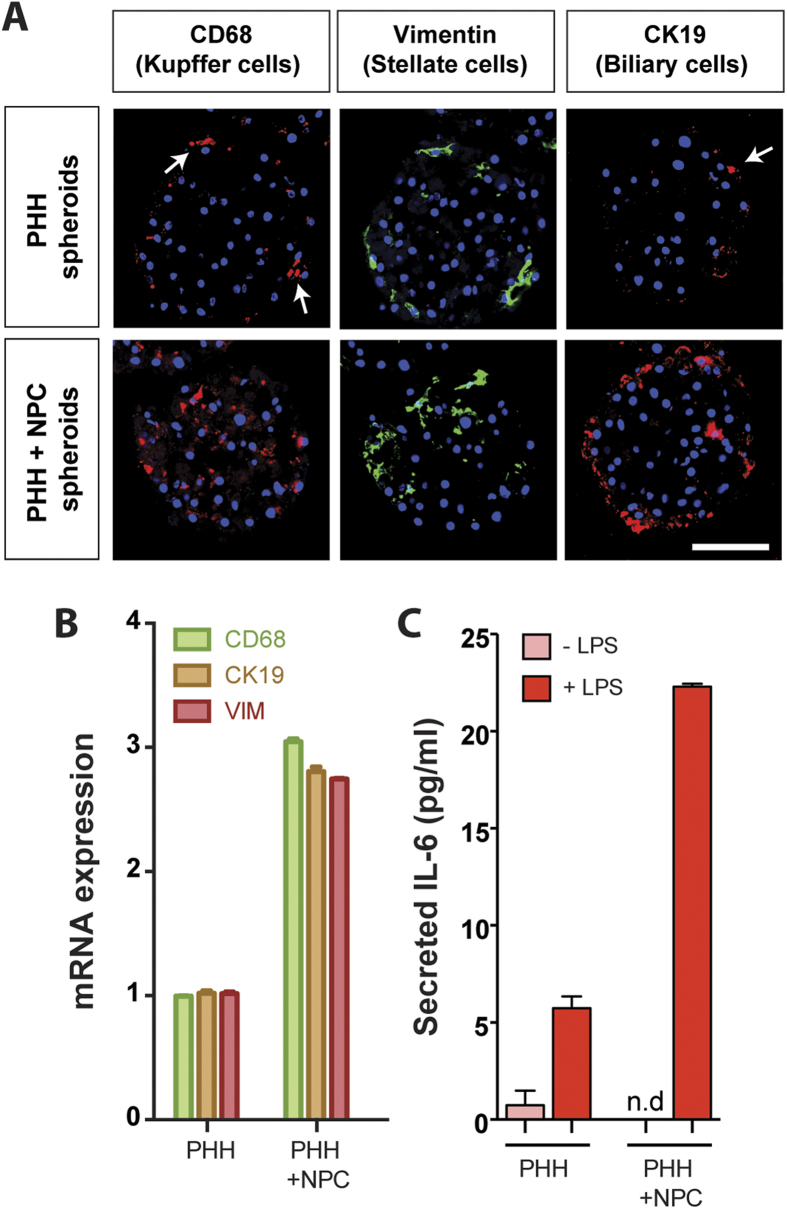
PHH spheroids can be successfully co-cultured with non-parenchymal Kupffer, stellate and biliary cells. Immunofluorescent stainings (**A**) as well as qPCR analyses (**B**) reveal the presence of Kupffer cells (CD68), stellate cells (vimentin) and biliary cells (CK19) in co-cultured spheroids at day 8 (bottom row). Note that some PHH preparations can already contain low numbers of NPCs (top row). (**C**) Co-cultured Kupffer cells were responsive to LPS-mediated activation as evidenced by elevated IL-6 secretion.

We investigated the possibility of introducing NPCs to the spheroid system to more closely reflect the *in vivo* composition of cells present in the liver. To this end, we co-cultured PHH with a mixture of stellate, Kupffer and biliary cells (Fig. 3). NPCs were clearly detectable after spheroid formation. While stellate and Kupffer cells were detected within the spheroid, biliary cells were located on the periphery, most likely as a consequence of adhesion-dependent cell sorting (Fig. 3A). Markers for these cell types were also detected at the mRNA level (Fig. 3B). Importantly, even after prolonged culture periods NPCs present in the hepatocyte preparation remained detectable thus indicating that the culture conditions presented here are compatible with long-term NPC viability and that the number of NPCs in relation to the number of PHH remains constant (Supplementary Figure S1). Furthermore, LPS treatment resulted in a substantial increase in IL-6 secretion in the co-culture spheroids compared to the PHH spheroids (Fig. 3C), confirming Kupffer cell activity in the spheroids. In addition, even without supplementing the PHH spheroids with heterologous NPCs, we observed that different preparations of cryopreserved hepatocytes contained low numbers of NPCs, which were also present in the PHH spheroids.

### PHH spheroids remain functional and metabolically active

**Figure 4:**
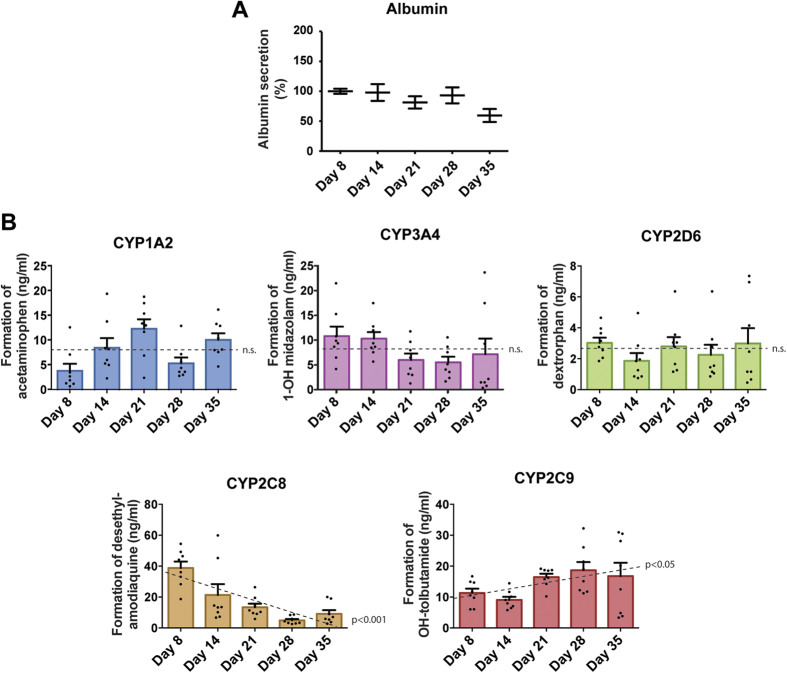
PHH cultured as spheroids remain metabolically active for at least 5 weeks in culture. (**A**) Albumin secretion normalized to spheroid volumes during long-term spheroid culture (n = 15 spheroids from 3 donors per time point). (**B**) CYP-dependent metabolic activity of PHH spheroids over 35 days. PHH spheroids were exposed to a cocktail of 5 CYP substrates and the resulting metabolites were analysed via LC-MS/MS (n = 8 spheroids per time point). No changes in rate of drug metabolism were detected for CYP1A2, CYP2D6 and CYP3A4 over the course of 5 weeks (n.s. corresponds to p > 0.05, F-test), whereas CYP2C8 and CYP2C9 activities were found to be significantly decreased (p < 0.001) or increased (p < 0.05) respectively.

To assess whether hepatocyte-specific functions were maintained in the PHH spheroids during prolonged culture, albumin secretion was analyzed and found to remain stable during prolonged culture (p = 0.51, F-test, Fig. 4A). The role of drug metabolism and the likely role of chemically-reactive metabolites in the toxicity of a number of compounds^20^ led us to investigate the activity of drug-metabolising enzymes in PHH spheroid culture over time. The PHH spheroids were treated with a cocktail of substrates for 5 major CYPs and formation of metabolites was monitored by LC-MS/MS (Fig. 4B). Activities of CYP1A2, CYP2D6 and CYP3A4 did not change significantly during 5 weeks of culture (p > 0.05 for all). In contrast, CYP2C8 activity reduced gradually and substantially to 23.5% ± 6% SEM after 5 weeks, whereas CYP2C9 activity increased (p < 0.05). Overall, the hepatic functions assessed in PHH spheroids were stable, indicating suitability for chronic drug toxicity assays.

### PHH spheroids are a suitable system to study chronic DILI

**Figure 5:**
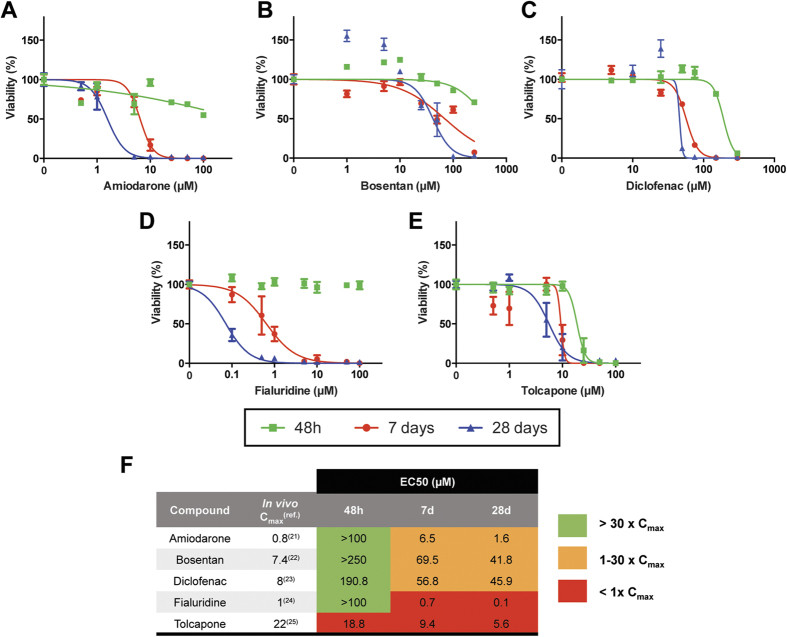
PHH spheroids support chronic toxicity assays. PHH spheroids were treated with amiodarone (**A**) bosentan (**B**) diclofenac (**C**) fialuridine (**D**) or tolcapone (**E**) every second day and viability was determined at 48 h, 8 days and 28 days by measuring cellular ATP content (n = 5–6 spheroids per concentration and time point). (**F**) Notably, EC_50_ values for all compounds decreased following long-term treatment (Fold changes in EC_50_ vs. 48 h: for amiodarone: 15.4-fold (8 d) and 62.5-fold (28 d); for bosentan: 3.6-fold (8 d) and 6.0-fold (28 d); for diclofenac: 3.4-fold (8 d) and 4.2-fold (28 d); for fialuridine: 142.9-fold (8 d) and 1000-fold (28 d); for tolcapone: 2-fold (8 d) and 3.4-fold (28 d)). Safety margins were calculated in order to relate the observed EC_50_ values to the physiological plasma C_max_ values observed *in vivo*.

To investigate whether PHH spheroids can be used to study DILI in a more chronic setting, the toxicity of 5 hepatotoxins during up to 4 weeks of drug treatment was assessed. Thus, PHH spheroids were dosed every 2 days with amiodarone, bosentan, diclofenac, fialuridine and tolcapone, and viability was determined after 48 h, 8 and 28 days. For all 5 hepatotoxins, prolonged exposure led to increased toxicity and a reduction in the EC_50_ values by up to 1000-fold, approaching clinically relevant concentrations (Fig. 5). This difference was particularly evident between 48 h and 8 days. The most striking increase in toxicity during prolonged treatment was seen for fialuridine where no toxicity was observed after 48 h, but the EC_50_ decreased to 100 nM after 4 weeks. This illustrates the potential of the PHH spheroids to detect potential hepatotoxicity for compounds previously negative in all human *in vitro* systems tested^17^. The potential for human hepatotoxicity was estimated by calculating safety margins, in which the EC_50_ values obtained in the spheroid system were related to the human plasma C_max_ of each compound (amiodarone:^21^, bosentan:^22^, diclofenac:^23^, fialuridine:^24^, tolcapone:^25^). In the case of tolcapone the EC_50_ values decreased with prolonged exposure. However the PHH spheroids were highly sensitive and the EC_50_ values were lower than the reported *in vivo* C_max_ values already after acute dosing. Yet, in the absence of intracellular data, C_max_ values can only approximate relevant concentrations, which might, at least partially, explain the particularly low EC_50_ values observed *in vitro*.

For the other compounds we tested, hepatotoxicity would not have been identified using a short-term 48 h exposure (Fig. 5F, green shading) but was clearly detected after prolonged exposures for 8 and 28 d (Fig. 5F, orange and red shading) using a safety margin of 30^26^,^27^. Our findings therefore suggest that the PHH spheroid system is a suitable model for predicting long-term *in vitro* toxicity.

### Liver pathologies such as steatosis and cholestasis can be replicated in PHH spheroids

**Figure 6:**
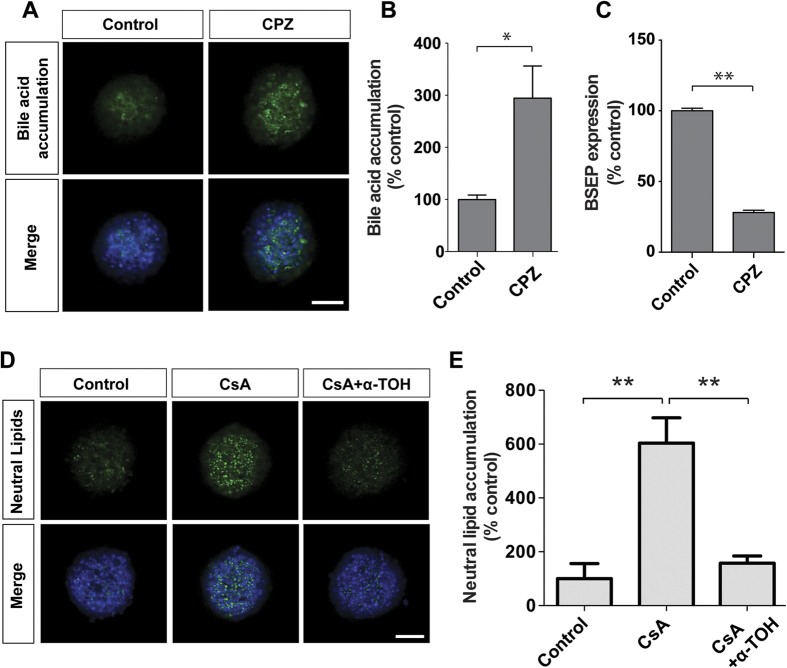
PHH spheroids as a model for cholestatic and steatotic disease. (**A–C**) Treatment with the cholestatic drug chlorpromazine (CPZ, 5 μM) caused significant accumulation (n = 3, p = 0.03) of the fluorescently-labelled bile acid derivative tauro-nor-THCA-25-DBD, which was associated with down-regulation of BSEP mRNA (**C**). (**D**,**E**) Cyclosporine A (CsA, 30 μM), a known inducer of steatosis *in vivo*^45^ increased levels of neutral lipids (n = 3, p = 0.003). Strikingly, cells were fully protected by co-exposure with the anti-oxidant α-tocopherol (α-TOH, 10 μM) (n = 3, p = 0.009). Bile acid and lipid accumulation were quantified and normalized to spheroid size using CellProfiler software. All scale bars = 100 μm. *indicates p < 0.05, **indicates p < 0.01.

To assess whether the PHH spheroids represent a suitable system for studies of specific liver pathologies, it was determined whether probe drugs could induce cholestasis and steatosis. Accordingly, the PHH spheroids were first exposed to chlorpromazine, and significant bile acid accumulation was detected, indicating impaired bile acid transport, a hallmark of cholestasis (Fig. 6A,B). In line with these findings, we observed that chlorpromazine repressed bile salt export pump (BSEP) mRNA expression (Fig. 6C), highlighting a potential mechanism for this accumulation^28^. Furthermore, cyclosporine A treatment resulted in significant enrichment of neutral lipids, indicative of steatosis (Fig. 6D,E) a process strongly inhibited by the simultaneous presence of the antioxidant α-tocopherol^29^, thus suggesting that the PHH spheroid system can reproduce steatotic pathologies *in vitro* and, moreover, is suitable to study underlying disease mechanisms as well as for candidate anti-steatotic drug screening.

### The PHH spheroid system is a useful model system for viral hepatitis

**Figure 7:**
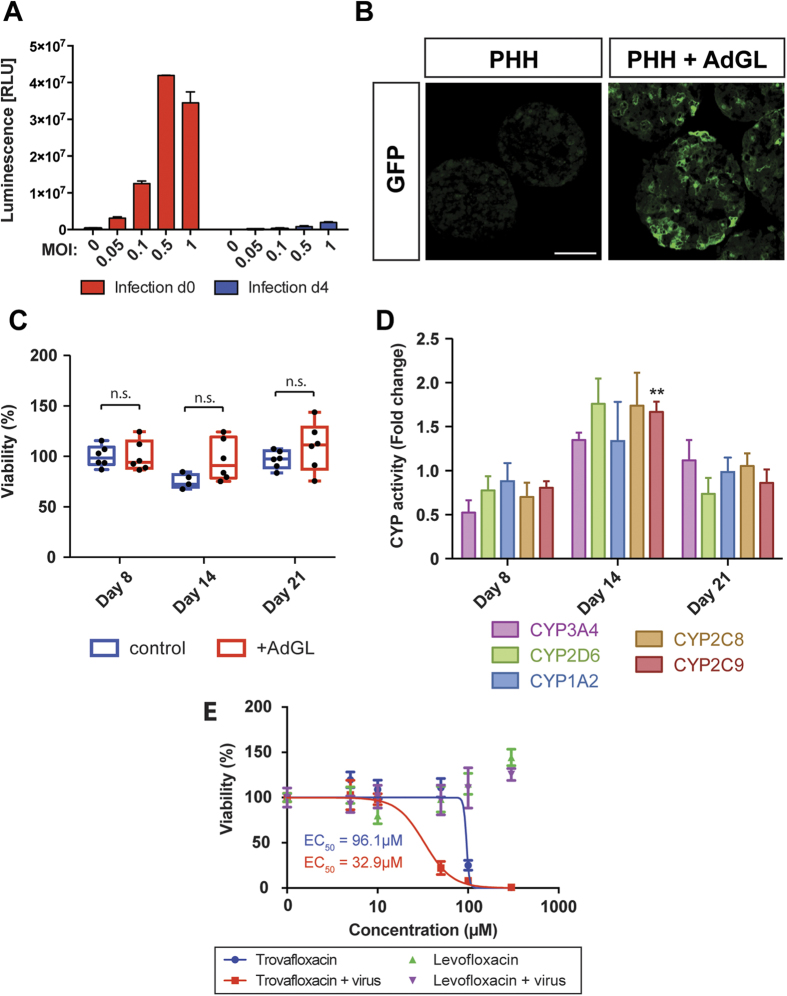
PHH spheroids form efficiently from virus infected hepatocytes. (**A**) Infection of PHH with recombinant adenovirus expressing GFP and luciferase (AdGL) was most effective if performed upon seeding (red columns; d 0) rather than when cells had aggregated into spheroids (blue columns; d 4), presumably due to the compactness of the spheroid at day 4 which hampers efficient penetration of the virus. All further experiments were performed using MOI of 0.1 due to reduced viability at higher MOIs. (**B**) GFP expression was observed throughout the spheroid. Scale bar = 100 μm. (**C**) Cell viability as determined by ATP measurements in virus-infected spheroids was not affected at MOI = 0.1. (p > 0.05 for all time points analyzed). (**D**) Enzyme activities of 5 key CYP enzymes (n = 8 spheroids per time point). Only CYP2C9 activity after 14 d differed significantly (Benjamini-Hochberg correction, FDR = 0.1) compared to uninfected spheroids (compare Fig. 3). (**E**) Viral infection sensitized cells to trovafloxacin hepatotoxicity while the non-hepatotoxic analogue levofloxacin showed no toxicity. **indicates p < 0.01, n.s. indicates p > 0.05.

Next, it was evaluated whether the PHH spheroid system can be used to mimic viral hepatitis *in vitro*. The PHH spheroids were efficiently infected by recombinant adenovirus prior to, but not after aggregation, as judged by quantifying the luminescence of spheroids infected at either day 0 upon seeding or day 4 (Fig. 7A). Introduction of the virus during seeding resulted in extensive GFP expression throughout the spheroid (Fig. 7B). The amount of virus was carefully titrated in order to ensure that spheroids were efficiently infected as judged by sufficient levels of luciferase expression (Fig. 7A) while being fully viable (Fig. 7C, p > 0.05 for every time point analyzed) and functional (Fig. 7D) even in long-term experiments.

Treatment of infected PHH spheroids with trovafloxacin, whose hepatotoxicity is amplified by inflammatory stimuli^30^, enhanced toxicity by 2.9-fold (p < 0.001), indicating virus-mediated inflammatory responses, whereas no toxicity of the non-hepatotoxic analogue levofloxacin was observed in both non-infected and infected spheroids.

## Discussion

*In vitro* cellular systems are commonly used to mimic the human liver in order to perform functional and toxicological studies. The HepG2 and HepaRG cell lines constitute routinely used models, but the phenotype of these cell lines differs substantially from PHH^31^,^32^. PHH are considered the gold standard, but when maintained in simple 2D monolayer cultures, cells rapidly de-differentiate resulting in the loss of liver-specific functions^3^. In sandwich culture, this de-differentiation process is delayed but not prevented^12^,^33^ and it is unclear how the sandwich configuration affects the PHH phenotype during long-term cultures. In 3D PHH spheroid culture, the complex structure of the human liver is recapitulated, resulting in improved viability and various liver-specific functions^14^,^15^. To date, PHH spheroids have to a great extent been generated using bioreactors^7^,^14^. However, limitations of these systems include the inability to control spheroid size, difficulties in handling as well as the need for high cell numbers. Scaffold-free spheroid formation in which spheroid size is controlled has currently only been performed using a hanging-drop system^15^. However, an extensive characterization of this system for studies of e.g. liver function, mechanisms of liver diseases, drug-induced liver injury (DILI) is lacking.

Unlike in previous studies^14^,^15^, the PHH spheroids presented here are maintained in chemically-defined, serum-free conditions. This is particularly important for toxicological studies, in which the binding of drugs and/or drug metabolites to serum proteins may confound the interpretation of viability data. Furthermore, the absence of growth factors from the media prevents the activation of extraneous cellular signaling pathways. The PHH spheroids described here were found to closely resemble intact liver tissues at the proteome level, using unbiased whole proteome profiling. In comparison to 2D monolayer cultures originating from the same livers we found that a multitude of important metabolic pathways were misregulated in 2D, including glycolysis, gluconeogenesis and the γ-glutamyl cycle, a key pathway in maintaining redox homeostasis^34^. Interestingly, it was found that even as early as after 24 h, the molecular signature of 2D monolayer-cultured hepatocytes revealed substantial differences compared to liver in the overall proteome as well as in ADME proteins in particular (Fig. 1B–F). In contrast, we did not detect significantly misregulated pathways in the corresponding 3D spheroid cultures. Moreover, the hepatic phenotype is maintained during long-term culture as evidenced by sustained ATP levels (Fig. 2F), albumin secretion (Fig. 4A) and overall stable CYP enzyme activity (Fig. 4B).

The preserved hepatic phenotypes and long-term functionality in the PHH spheroids allowed us to test their versatility in chronic toxicity assays. Long-term dosing enhanced the sensitivity of PHHs to a panel of 5 hepatotoxins (Fig. 5), thereby reflecting the delayed onset of many DILI reactions seen *in vivo*^35^. We observed significant reduction in the EC_50_ values for all compounds tested which was particularly noticeable between 48 h and 8 days (Fig. 5F), thus reaching toxicity *in vitro* at clinically relevant concentrations. This was most prominent for fialuridine, for which cytotoxicity was exclusively detected upon long-term dosing (EC_50_ > 100 μM at 48 h vs. 0.1 μM at 28 days). In clinical trials, 7/15 patients developed severe hepatotoxicity, several weeks after beginning fialuridine treatment, five of whom died^17^. Despite this, no indication of fialuridine hepatotoxicity was observed in any pre-clinical testing. A step forward to the elucidation of its hepatotoxicity was found in chimeric TK-NOG mice where fialuridine induced hepatotoxicity was obtained in mice carrying human hepatocytes^36^. Here we provide a model system that is capable of predicting fialuridine toxicity *in vitro*, thus indicating that the PHH spheroid model can provide a powerful tool to predict and to dissect the mechanisms underlying compound toxicity during pre-clinical drug development stages.

Inter-individual differences in drug response can cause severe ADRs which are a major cause of withdrawal of drugs from the market^37^. To date, only 15–30% of these inter-individual differences in response to pharmacological treatment can be explained^38^. Mechanisms for the remaining fraction of ADRs are missing due to the lack of suitable hepatic model systems that can successfully reflect inter-individual variability *in vitro*. Recent studies reported that inter-individual differences in CYP metabolism could be preserved using iPS-derived hepatocyte-like cells (HLCs) that were derived from PHH^39^. While interesting, this protocol has intrinsic limitations as it is financially demanding, time consuming and HLCs do not reach full differentiation. Thus, here we considered whether PHH cultured as spheroids recapitulate inter-individual variability. Importantly, when whole proteomes were assessed, hierarchical clustering resulted in livers and 3D PHH spheroid cultures from the same donor grouping more closely together than livers from different individuals, thus indicating that inter-individual variation is preserved in the spheroid cultures (Fig. 1D). Therefore, the PHH spheroid system can potentially be used to study hepatocytes with specific genotypes of interest, which are pertinent to hereditary diseases or genetic variations in metabolizing enzymes with importance in drug response. In addition, by using selective antagomiR or siRNA-based knock-down of specific gene products in the spheroids, the role of specific genes in e.g. liver disease and in response to drug treatment can be studied (Supplementary Figure S2). Here we show the effective delivery of antagomiRs to the PHH spheroids, and an increase in CYP2C8 expression when miR103 is specifically targeted^40^.

In addition, the potential of the PHH spheroids as a model system for liver disease was assessed, focusing on cholestasis, steatosis and hepatitis, liver pathologies that can be the consequence of several factors, including exposure to certain drugs. We found that bile acid accumulation, the major hallmark of cholestasis could be reproduced in the PHH spheroid system after exposure to chlorpromazine (Fig. 6A,B). When proteins important for bile transport were analyzed in detail in PHH spheroids, it was found that the bile acid pump BSEP (p = 0.36) and the bile exporter MRP2 (p = 0.54) were not significantly altered compared to liver tissue suggesting that the molecular machinery involved in bile acid circulation is preserved in spheroid culture. Chlorpromazine caused a major downregulation of BSEP mRNA (Fig. 6C), which indicates that the mechanisms attributed to chlorpromazine-induced cholestasis are reflected in the spheroid system^28^. To assess the suitability of PHH spheroids as a steatotic model system, hepatocytes were treated with cyclosporine A and a rapid enrichment of lipids was detected in spheroids (Fig. 6D,E). Importantly, the lipid accumulation could be prevented by co-treatment with α-tocopherol^29^. Thus, PHH spheroids constitute promising *in vitro* systems for the evaluation of drug-induced cholestasis and steatosis and enable investigations of underlying disease mechanisms, or the pharmacological action of new drugs. In addition, it was found that PHH spheroids can be readily infected with virus which renders them potentially useful for the study of liver function and toxicity in the context of viral infection and inflammation. Underlying viral infection is thought to be a significant risk factor for DILI, and both HIV and HBV infections have been associated with increased susceptibility to certain drug toxicities^39^,^41^. Inflammagens such as LPS and viruses can render hepatocytes more sensitive to cellular stress through toll-like receptor signalling and the production of inflammatory cytokines. In our system, viral infection enhanced trovafloxacin-induced toxicity *in vitro* thereby reproducing inflammation-mediated sensitization phenomena observed *in vivo*^30^,^41^.

In summary, PHH spheroids in the culture system described here were found to closely resemble *in vivo* human liver with respect to their proteomes, morphological and molecular phenotypes as well as inter-individual variability. They remain viable and functional over prolonged culture periods, enabling chronic exposure studies and facilitating drug screening for chronic DILI. In addition, we showed that the PHH spheroid system is very versatile and can be used to study a variety of liver diseases and DILI risk factors.

## Methods

### Materials

Cell culture medium, medium supplements, and compounds were obtained from Sigma-Aldrich (Sweden) or Life Technologies (Sweden) unless stated otherwise.

### PHH spheroid cultures

Cryopreserved PHH (obtained from KalyCell, France or Bioreclamation IVT, USA) or fresh hepatocytes (obtained from patients subject to liver resections at Huddinge University Hospital, Stockholm, Sweden) were used for formation of spheroid cultures. Hepatocytes obtained from patient livers were isolated as previously described^42^. Use of the liver specimens for this purpose was approved by the Ethics Committee at Karolinska Institutet (Regionala etikprövningsnämnden i Stockholm) and studies were carried out in accordance with the approved guidelines. Written informed consent was obtained from all donors of liver material. Cells were seeded into ultra-low attachment 96-well plates (Corning) at 1,500 viable cells per well and subsequently centrifuged at 100 × *g* for 2 min. Cells were seeded in 100 μl Williams E medium supplemented with 2 mM L-glutamine, 100 units/ml penicillin, 100 μg/ml streptomycin, 10 μg/ml insulin, 5.5 μg/ml transferrin, 6.7 ng/ml sodium selenite, 100 nM dexamethasone, and 10% FBS. Spontaneous self-aggregation of the hepatocytes initiated spheroid formation. From day 4 or 5 after seeding, when the spheroids were sufficiently compact, 50% of the medium was exchanged daily for serum-free medium. Spheroids were maintained in serum-free medium until day 35, with a medium change every 48–72 h.

### Co-culture spheroids

When used, co-cultures of cryopreserved PHH and non-parenchymal cells (NPCs; Bioreclamation IVT) originating from different donors were thawed and seeded at a ratio of 2:1 (PHH:NPC) and 2,000 viable cells per well into ultra-low attachment plates. Functionality of Kupffer cells was assessed by measuring lipopolysaccharide (LPS)-induced interleukin 6 (IL-6) secretion. LPS was added at a concentration of 10 μg/ml and supernatants were collected after 48 h. IL-6 levels were determined via ELISA (Thermo Scientific).

### Proteomics

Freshly isolated PHH were compared to liver tissue, 3D PHH spheroids after aggregation (7 d) and cells cultured as 2D monolayers (24 h and 7 d) from the same donors. Cells were washed, scraped and pelleted in phosphate buffer (pH 7.4). Subsequently, cells were resuspended in a volume of 0.5 M TEAB/0.1% SDS equivalent to cell pellet volume. Liver samples (50–100 mg) were homogenised in 0.5 M TEAB/0.1% SDS using a Mixer Mill 220 (Retsch, Haan, Germany). Cell and liver samples were subjected to one freeze-thaw cycle, sonicated and centrifuged. 100 μg protein/sample were denatured, reduced and treated with methyl methanethiosulfonate according to the manufacturer’s protocol (Sciex, Framingham, MA, USA), before being labelled with isobaric tags for absolute and relative quantification (iTRAQ), pre-fractionated by cation exchange chromatography and analysed on a Triple TOF 5600 (Sciex) as previously described^43^. Samples from each donor were analysed in a single iTRAQ run. Data were searched using ProteinPilot 4.2 and the Paragon algorithm (Sciex) against the SwissProt database and a reversed decoy database and only proteins lying within a 1% global false discovery rate (FDR) were taken forward for analysis.

### Statistical analysis

Principle component analyses were computed using Qlucore Omics Explorer 3.1. To this end, only proteins whose abundances were significantly different (p < 0.05, F-test) between the different time points were considered. Hierarchical clustering was performed on mean-centered sigma-normalized protein levels using maximum linkage in Qlucore Omics Explorer 3.1. Gene set enrichment analysis was performed using Ingenuity Pathway Analysis (Qiagen, Sollentuna, Sweden).

Deviations from linearity were calculated using F-tests (Prism, GraphPad Software, USA). For two-group comparisons, heteroscedastic Student’s t-tests were calculated in Excel (Microsoft). When numbers of tests exceeded 10, multiple testing corrections were employed using the Benjamini-Hochberg procedure with false discovery rates of 5% or as indicated.

### Immunohistochemistry

Spheroids were collected, fixed with 4% formaldehyde for 24 h, cryoprotected in 30% sucrose and embedded in Tissue-Tek OCT compound (Sakura, The Netherlands). Spheroid cryosections (8 μm) were stained for CYP3A4 (PAP011, 1:5,000, Cypex Limited, United Kingdom), albumin (sc51515, 1:200, Santa Cruz, USA), E-cadherin (13–1700, 1:300, Thermo Scientific), MRP2 (ab3373, 1:100, Abcam, United Kingdom), CK19 (sc6278, 1:100, Santa Cruz), CD68 (sc70761, 1:50, Santa Cruz), Cleaved caspase-3 (9661, 1:500, Cell Signaling Technology, USA) and Vimentin (ab92547, 1:200, Abcam). CellProfiler software was used to quantify marker staining relative to spheroid size.

### Assesment of viability and albumin secretion

Cell viability was assessed by determining the ATP content of single PHH spheroids with the CellTiter-Glo Luminescent Cell Viability Assay (Promega, Sweden). At the same time-points, cell culture medium was collected and albumin concentrations were determined by ELISA (Bethyl laboratories, USA). Absolute ATP and albumin values were normalized to spheroid volumes as determined from bright field imaging to compensate for compaction.

### Determination of CYP450 enzyme metabolic activity

PHH spheroids were incubated with a mixture of probe substrates, i.e. midazolam (CYP3A4, 10 μM), dextromethorphan (CYP2D6, 15 μM), phenacetin (CYP1A2, 100 μM), amodiaquine (CYP2C8, 10 μM) and tolbutamide (CYP2C9, 100 μM) and supernatants were collected after 24 h. Formed metabolites (1-OH-midazolam, dextrorphan, acetaminophen, desethyl-amodiaquine and OH-tolbutamide) were quantified by LC/MS-MS. Deuterated 1-OH midazolam was added as an internal standard. For all metabolites but OH-tolbutamide, a mobile phase starting with solvent A (5% acetonitrile, 0.1% CH_3_OOH) shifting to solvent B (95% acetonitrile, 0.1% CH_3_OOH) using a linear gradient was pumped at a flow rate of 0.6 ml/min. Separation was carried out using an Acquity UPLC C18 column and eluted fractions were directly passed through a Xevo TQ-S tandem mass spectrometer (both from Waters Corp., Milford, MA, US) equipped with an electrospray ionization source operating in the positive ion mode. For OH-tolbutamide, a mobile phase starting with solvent A (4% methanol, 0.1% formic acid) shifting to solvent B (95% methanol, 0.1% formic acid) shifting back to solvent A was used and the tandem mass spectrometer was operated in the negative ion mode. Acquired data were processed with Thermo Xcaliber software (Thermo Scientific, Waltham, MA, US). All results are expressed as measurements from single spheroids. An overview of mass transition for CYP450 probe substrates is given in Supplementary Table 1.

### Toxicity assays

Stock solutions of amiodarone, bosentan (Sequoia, St. Louis, MO, USA), diclofenac, fialuridine (Carbosynth, Berkshire, UK) and tolcapone were diluted in serum-free medium to a maximal final DMSO concentration of 0.4%. Treatment was started on day 8 with repeated dosing of PHH spheroids every second day in serum-free medium as indicated. Cell viability was assessed by determining the ATP content as indicated above. EC_50_ values were determined using GraphPad Prism (GraphPad Software, USA).

### Bile acid accumulation

PHH spheroids were dosed with 5 μM chlorpromazine every second day for 1 week. For the final exposure, 20 μM tauro-nor-THCA-25-DBD (Genomembrane Company Ltd., Yokohoma, Japan) was added. Bile acid accumulation was quantified and normalized to spheroid size using CellProfiler software.

### RT-qPCR analysis

Total RNA was isolated using Qiazol lysis reagent (Qiagen). RNA was reverse-transcribed into cDNA using SuperScript III reverse transcriptase and RT-qPCR analysis was performed using a TaqMan Universal mix on a 7500 Fast Real-Time PCR system.

### Neutral lipid staining

PHH spheroids were treated with 30 μM cyclosporine A (CsA) and/or 10 μM α-tocopherol (α-TOH) as indicated. After 48 h, spheroids were fixed and incubated with HCS LipidTOX Green neutral lipid stain (1:500). CellProfiler software was used to quantify neutral lipid staining.

### Viral infection of spheroids

A recombinant adenovirus (AdGL) expressing green fluorescent protein (GFP; described in ref. 44) was added during PHH spheroid seeding at a multiplicity of infection (MOI) of 0.1. PHH were seeded into GravityPLUS hanging-drop 96-well plates (InSphero, Zurich, Switzerland) at 1,500 viable cells per well. Spheroids were transferred to Gravity TRAP 96-well plates (InSphero) on day 7. Culture conditions were identical to the conditions for spheroids formed in ultra-low attachment plates.

### Confocal imaging

All fluorescent images were acquired using an LSM710 confocal microscope (Zeiss, Germany). Images were processed with ZEN lite 2012 analysis software (Zeiss, Germany).

## Additional Information

**How to cite this article**: Bell, C. C. *et al.* Characterization of primary human hepatocyte spheroids as a model system for drug-induced liver injury, liver function and disease. *Sci. Rep.* **6**, 25187; doi: 10.1038/srep25187 (2016).

## Supplementary Material

Supplementary Information
