# Supplementary material for: Characterization of primary human hepatocyte spheroids as a model system for drug-induced liver injury, liver function and disease
Source: Sci Rep. 2016 May 4;6:25187. doi: 10.1038/srep25187 (PMC4855186; doi:10.1038/srep25187)
Supplement: Supplementary Information [file srep25187-s1.pdf]

# **Characterization of primary human hepatocyte spheroids as a model system for drug-induced liver injury, liver function and disease**

Catherine C. Bell[1§], Delilah F. G. Hendriks[1§], Sabrina M. L. Moro[1§], Ewa Ellis[2], Joanne Walsh[3], Anna Renblom[1], Lisa Fredriksson Puigvert[1], Anita C. A. Dankers[4], Frank Jacobs[4], Jan Snoeys[4], Rowena L. Sison-Young[3], Rosalind E. Jenkins[3], Åsa Nordling[1], Souren Mkrtchian[1], B. Kevin Park[3], Neil R. Kitteringham[3], Christopher E. P. Goldring[3], Volker M. Lauschke[1], Magnus Ingelman-Sundberg[1]*

[1] Department of Physiology and Pharmacology, Section of Pharmacogenetics, Karolinska Institutet, Stockholm, Sweden

[2] Department of Clinical Science, Intervention and Technology, Karolinska University Hospital Huddinge, Karolinska Institutet, Stockholm, Sweden

[3] MRC Centre for Drug Safety Science, Department of Molecular and Clinical Pharmacology, Sherrington Buildings, Ashton Street, University of Liverpool, UK.

[4] Janssen Pharmaceutical Companies of Johnson & Johnson, Department of Pharmacokinetics, Dynamics and Metabolism, Beerse, Belgium

[§] These authors contributed equally to the manuscript.

* Corresponding author: Dr. Magnus Ingelman-Sundberg, Karolinska Institutet, Department of Physiology and Pharmacology, Section of Pharmacogenetics, Nanna Svartz Väg 2, 17177 Stockholm, Sweden, Phone: +46 8524 877 35, E-mail: magnus.ingelman-sundberg@ki.se

**Supplementary Information**

Included Supplementary Material:

Supplementary Figures 1-2

Supplementary Table 1

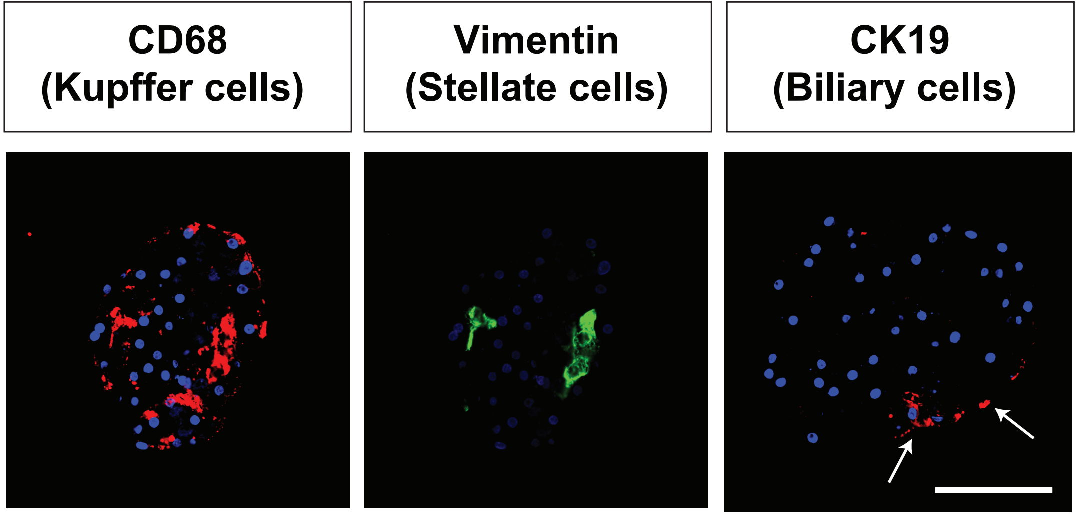


**Supplementary Figure S1: The presence of non-parenchymal cells in PHH spheroids persists in long-term culture.** Immunofluorescent stainings reveal the presence of non-parenchymal Kupffer cells (CD68), stellate cells (vimentin) and biliary cells (CK19) in PHH spheroids after 21 days of culture.

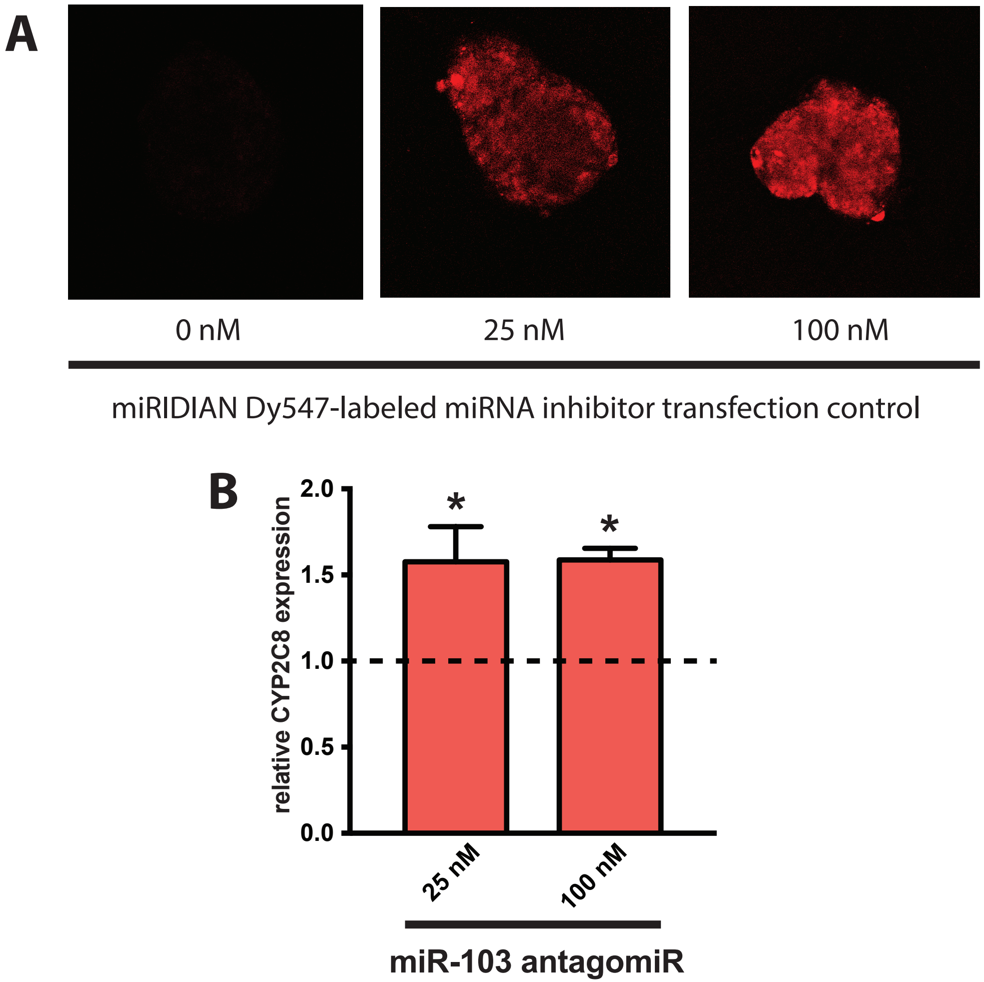


**Supplementary Figure S2: Inhibition of miR-103 results in increased expression of its target gene *CYP2C8***. (A) PHH spheroids were transfected with the fluorescent Dy547-labeled miRNA transfection control reagent to monitor the delivery of miRIDIAN antagomiRs. (B) Spheroids were transfected with miR-103 antagomiR as described in Materials and Methods. Total RNA was isolated from spheroids at day 7 after transfection. *Cyp2c8* mRNA levels were quantified by real time PCR using the corresponding TaqMan probe. Data are normalized to the housekeeping gene TBP and presented ±SEM relative to the *CYP2C8* expression in the cells transfected with the control antagomiR. * corresponds to $p<0.05$.

**Supplementary Table 1.** Overview of mass transition for CYP450 probe substrates

| | Retention time (min) | Parent Mass | Collision energy (V) | Fragment |
|---|---|---|---|---|
| OH-Tolbutamide | 0.80 | 285 | 18 | 186 |
| Acetaminophen | 0.80 | 152 | 15 | 110 |
| Desethyl-Amodiaquine | 0.95 | 328 | 18 | 283 |
| Dextrorphan | 1.05 | 258 | 13 | 201 |
| 1-OH Midazolam | 1.35 | 342 | 15 | 324 |
| 1-OH-Midazolam-D4 (IS) | 1.35 | 346 | 15 | 328 |
